# Development and Utilization of 3D Printed Material for Thoracotomy Simulation

**DOI:** 10.1155/2018/9712647

**Published:** 2018-11-15

**Authors:** Evan Yates, Roger Chirurgi, Frosso Adamakos, Rania Habal, Rajnish Jaiswal, Hossein Kalantari, Getaw Worku Hassen

**Affiliations:** ^1^Western University of Health Sciences, USA; ^2^Samuel Johnson School of Management Cornell Tech, USA; ^3^NYMC, Metropolitan Hospital Center, Department of Emergency Medicine, New York, NY, USA

## Abstract

Medical simulation is a widely used training modality that is particularly useful for procedures that are technically difficult or rare. The use of simulations for educational purposes has increased dramatically over the years, with most emergency medicine (EM) programs primarily using mannequin-based simulations to teach medical students and residents. As an alternative to using mannequin, we built a 3D printed models for practicing invasive procedures. Repeated simulations may help further increase comfort levels in performing an emergency department (ED) thoracotomy in particular, and perhaps this can be extrapolated to all invasive procedures. Using this model, a simulation training conducted with EM residents at an inner city teaching hospital showed improved confidence. A total of 21 residents participated in each of the three surveys [(1) initially, (2) after watching the educational video, and (3) after participating in the simulation]. Their comfort levels increased from baseline after watching the educational video (9.5%). The comfort level further improved from baseline after performing the hands on simulation (71.4%).

## 1. Background

Medical simulation is a widely used training modality, especially for procedures that are difficult or rarely seen in practice. Most EM residency programs in the United States use some sort of medical simulation in their curriculum. The use of simulation for education has increased over the years and most EM programs primarily use mannequin-based simulations to teach medical students and residents. Simulation training has contributed significantly to the education of not only medical residents, but also other health care professionals [1–10]. Simulated procedures help increase health care provider comfort and competency for future real-time encounters, help reinforce the step-by-step procedural skills that are developed through repetitive practice, and decrease anxiety and complication rates ultimately leading to better patient outcome [11–18]. A recent study by Bohnen et al. using a high-fidelity mannequin for ED thoracotomy showed improvement of surgical trainee's confidence [19].

Resident physicians typically practice ED thoracotomies primarily through the use of cadavers. While effective as a mode of ancillary teaching, cadavers are not readily available and are costly. An alternative way to practice ED thoracotomy is through self-made models that represent a more ideal setting for thoracotomy practice. This ideal setting includes having organs and body parts that are present in the proper location. These body parts include the heart, lungs, diaphragm, phrenic nerve, esophagus, ribs, intercostal muscles, blood vessels, and skin. This proposed 3D printed model contains most of these components and provides physicians an alternative, inexpensive way to gain practice in performing an ED thoracotomy. By increasing the frequency of practice procedures using simulations, it is assumed that overall level of comfort and competency will increase over time.

## 2. Materials and Methods

**Study setting:** The study was conducted in an urban EM residency program with a total of 24 residents during one of the weekly conference day reserved for simulation. Before the study participants were briefed about the study plan which included a brief description of emergency thoracotomy followed by a pretest survey followed by watching a selected ED thoracotomy video (https://www.thecgroup.com) that demonstrated step by step an ED thoracotomy on a high-fidelity simulation a mannequin. Participants completed a second survey after the video demonstration. At the end they participated in hands-on ED thoracotomy under the supervision of an attending physician followed by a third survey.

**Figure 1:**
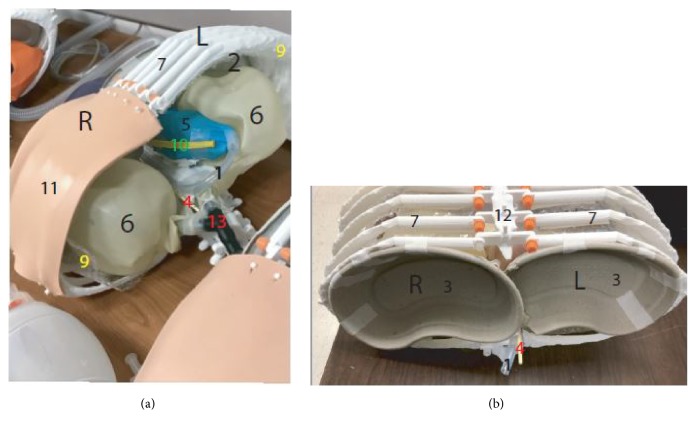
1= Aorta. 2= Chest cavity. 3= Diaphragm. 4= Esophagus. 5= Heart with pericardium. 6= Lung. 7= Rib. 8= Rib spreader. 9= Parietal pleura. 10= Phrenic nerve. 11= Skin with subcutaneous tissue and chest wall muscle. 12= Sternum. 13= Trachea with main bronchi. L= left side. R= right side.

**Figure 2:**
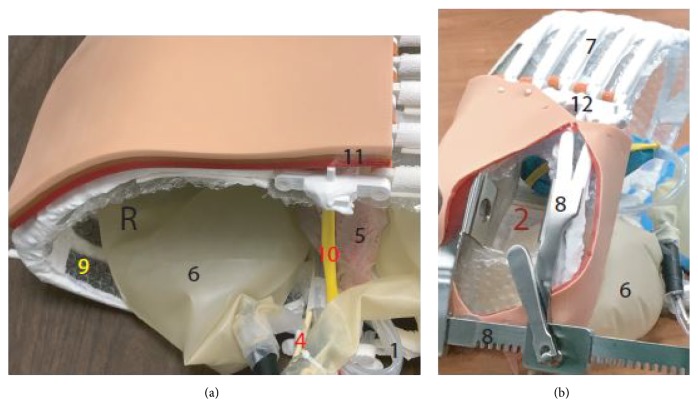
1= Aorta. 2= Chest cavity. 3= Diaphragm. 4= Esophagus. 5= Heart with pericardium. 6= Lung. 7= Rib. 8= Rib spreader. 9= Parietal pleura. 10= Phrenic nerve. 11= Skin with subcutaneous tissue and chest wall muscle. 12= Sternum. 13= Trachea with main bronchi. L= left side. R= right side.

**Study model:** A model consisting of 5 vertebrae, 10 ribs, and a sternum was created from anonymized images obtained from a computed tomography (CT) scan. The CT images were converted to a 3D printing file using Slicer and MeshMixer and further modified using TinkerCAD and the MakerBot software. The files were then 3D printed on a MakerBot Replicator+ 3D Printer. Only the bony structures were created using the 3D printer. The other items were acquired commercially and supplemented the model. The models were then overlaid with a custom repurposed suture board consisting of 3 layers skin, subcutaneous tissue, and muscle to increase likeness. A simulation training session was conducted using a model made of 3D printed ribs, vertebrae and sternum as well as plastic tubing representing the esophagus and aorta. In addition, the pericardium along with the phrenic nerve was simulated using a glove and a thin rubber as the phrenic nerve (Figures 1 and 2).

**Study participants:** All EM residents at an inner city teaching hospital were asked to participate voluntarily in the simulation training. A waiver was obtained from the corresponding institutional review board (IRB). Participants were given a pretest questionnaire to complete before the simulation. A second questionnaire was given to them after watching a didactic video about ED thoracotomy using high-fidelity manniquin. Finally, a third questionnaire was given after participating in the simulation training session using the 3D printed models under supervision by an attending EM physician. All questionnaires were anonymous and without participant identifying information. The questionnaire included aspects of procedure performing confidence, knowledge on the anatomy, and the ability to identify important structures. Comfort is defined as if they had to perform the procedure or identify structures or would be able to do it without any hesitation or second guessing. Residents were assigned a number according to their alphabetical name order. That number was indicated on each questionnaire to identify the resident's training level. Questionnaires 1, 2, and 3 represent the individual questionnaires (supplements 1-3). Figures 1 and 2 represent the individual components of the simulation kit.

## 3. Investigative Procedure

After the simulation training was completed and the questionnaires were collected, the level of comfort before and after watching the selected video and after participating in the simulation training were compared using visual analog scale (1-10). Additionally, the levels of ability to identify anatomical structures in the left chest cavity were compared before and after participating in the simulation. The level of comfort and the rate of improved comfort level after training were evaluated among all educational levels of residents. Responses were collected from the questionnaire and transferred to the Simulation Dataset in Excel. Descriptive statistic was conducted.

## 4. Results

A total of 21 residents participated in each of the three surveys. Eight residents (38.1%) were in the first year, 9 residents (42.9%) were in the second year and 4 residents (19%) were in the third year of EM-residency training. Of the 21, only 1 resident (4.8%) had performed an ED thoracotomy in the past, 5 residents (23.8%) had participated in the procedure, and 8 residents (38.1%) had observed the procedure. Seventeen residents (80.9%) had watched a video/videos of an ED thoracotomy previously. All residents reported knowledge of the thoracotomy-related major organs in the chest cavity. The level of comfort was arbitrarily divided into three categories [scores 1-4 (low), 5-7 (moderate), and 8-10 (high)]. When asked to indicate their initial comfort level in performing the procedure prior to both the video demo and the hands on simulation, 15 residents (71.4%) reported low confidence level, 5 residents (23.8%) reported moderate confidence level and 1 resident (4.8%) reported high confidence level. Additionally, in terms of identifying intrathoracic structures, 6 residents (28.6%) reported low confidence, 12 residents (57.1%) reported moderate confidence, and 3 residents (14.3%) report high confidence. After watching an educational video about ED thoracotomy, 10 residents (47.6%) reported low confidence level, 9 residents (42.9%) reported moderate confidence level, and 2 residents (9.5%) reported high confidence level in performing the procedure. After participating in the hands on ED thoracotomy simulation using the 3D printed model, 11 residents (52.4%) reported moderate confidence level and 10 residents (47.6%) reported high confidence level performing the procedure. After performing a thoracotomy using the 3D printed model, 6 residents (28.6%) reported moderate comfort level and 15 residents (71.4%) reported high confidence level if they were to perform the procedure in the future. The results of the study are summarized in Tables 1(a) and 1(b).

## 5. Discussion

Invasive procedures are an integral part of patient care in the ED, and are used as both diagnostic and therapeutic tool. Some procedures are performed frequently and others rarely, depending on the hospital's designation (level I or Level II) and the hospital's location (urban or rural). Familiarity with the steps of the procedure and prior experience are of great value for patient outcome and reduction in complications. Experience comes with frequent performance of a procedure and after having encountered difficulties and complications. Some procedures are performed very infrequently and thus knowledge and competency is gained primarily by practicing using alternative simulation methods such as animals, cadaver, video or mannequin-based simulations. Each option has advantages and disadvantages. Cadavers (particularly fresh frozen cadavers) and animals represent the optimal anatomical representation of organs and structures, but are very expensive and can only be used once. Video-based simulations lack the hands on experience and typically performed by an experienced practitioner demonstrating the procedure with each organ already identified systematically and with each step explained and performed without complications. While informative, this method makes the procedure appear easier to perform than it may actually be. Mannequin-based simulations allow frequent practices, but are expensive depending the type of procedure simulated [6, 9, 10, 12, 20–24].

Several studies have been conducted to evaluate the role of simulation in performing invasive procedures for healthcare providers of various specialties and training levels, as well as for medical students. Simulation based practice allows physicians to learn from their potentially fatal mistakes during simulation, which could possibly occur in real scenarios [13, 14, 25–27]. Simulation-based practices have been shown to improve procedural skills, decrease levels of anxiety, and help with identifying mistakes as well as allowing for debriefing opportunities to avoid similar mistakes in future. The effect of simulation practices vary based on the individual participant and skill level and prior experience of the participant. Simulation procedural practice is inherently different from video procedural demonstration or live observation. Hands-on simulations provides reality based scenarios with typical complications and challenges of live procedures. Challenges include preparation to perform the procedure, followed by the manual dexterity of handling of instruments and finally the performance of the procedure itself [2, 3, 12, 16–18, 28–33].

One of the observation made at the simulation setting was a higher level of anxiety in junior residents as compared to the senior residents. During our study we noted that even cutting of the skin with a scalpel appeared to become challenging. Additionally, some participants were slower and seemingly extra careful in cutting the skin as well as slower when spreading the ribs even though the procedure was performed on an inanimate object. One can imagine the difficulty when performing procedures on actual patients who are critically ill along with stress from multiple team members directly observing the provider.

Moreover, in video training all organs are in their ideal anatomical locations, each step of the procedure is explained and the procedure is performed by an experienced clinician. Organ identification is conducted in a simple manner without variations of the organ structures or positions. Mannequin, video-based procedures, and cadavers also do not reflect patients' individual physiological changes that occur in a trauma setting such as uncontrolled bleeding, inability to identify and control bleeding sources, presence of anatomical variations, spasms, or vomiting which can contribute to procedure related complications.

Thoracotomy is an infrequently performed procedure, and in order to obtain optimal performance, repeated simulation is essential. This procedure can be performed on cadavers, animals or mannequins, however those simulated models can cost up to $15,000-$20,000. We created a less expensive alternative for the purpose of this study, a 3D printed model that recreates the requisite organs and structures of the body and can be reused with minimal expenditure.

Our study showed that EM residents reported increased levels of confidence after watching an ED thoracotomy video performed on a mannequin. The comfort level further increased after they performed the procedure on our model.

A single exposure to a real or simulated procedure does not result in competency. This begs the question as to how many simulated procedures must be performed in order to gain competency. Every physician remembers their first experience in placing an intravenous (IV) line after watching other perform the procedure with ease and swiftness.

A study by Wong et al. demonstrated that participants required 5 attempts to perform simulated Circothyroidotomy in less than 40s, to become competent [34]. For all procedures, even as simple as placing IV line, the first attempt will bring many question to the performer: How do I open the kit? How should I hold the Angiocath? At what angle should I aim? How deep should I go? When do I stop? What do I do next? Is the patient in pain? What if I hit an artery or a nerve? And so forth. It is a nerve wracking experience, but with practice and experience, all these questions will be pushed into the back of the mind. If a simple procedure such as putting an IV creates anxiety and distress, performing a far more challenging procedure such as a thoracotomy is likely to lead to far greater anxiety and distress.

Gaining competency in performing thoracotomy may not be necessary for most specialties, but 3D printed models can be utilized in teaching diverse procedures such as central line placement, lumbar puncture, arterial line placement, paracentesis and thoracentesis to all medical students regardless of the specialty that they may choose upon graduation. Simulated procedures with 3D printed material are cost effective and reproducible and can be organized in a way to represent procedure-related challenges, complications, and abnormal patient anatomy. Exposing residents and medical students to repeated procedure simulation allows them to gain both confidence and procedural dexterity. It also helps learners in developing the critical thinking skills necessary to not only perform the actual procedure, but also to practice general patient safety measures such as identifying patients, confirming the correct procedure and the site, applying aseptic techniques, and more. Practicing this comprehensive approach can help reduce procedure-related infections, delays, patient pain or discomfort, and performing the wrong procedure on the wrong site, all ultimately leading to better patient outcomes and satisfaction.

In summary, our study demonstrated that simulation improved the level of comfort. More practice based simulation may improve the resident comfort level and decrease anxiety. A hands-on procedural course using a 3D printed model seems to be a viable and less costly alternative to other modes of hands on simulation.

## 6. Limitation

The study is limited by small sample size and it was conducted in one site only. In its current form the study did not have a follow-up after several simulations to appreciate the changes by repeated practice. We intend to organize several sessions and repeat the study to see if comfort level of residents in performing the procedure improves significantly.

Finally, previous experience from direct participation or observation as well as the number of timed they watched other educational video for ED thoracotomy was not considered for analysis or comparison.

## 7. Conclusion

Simulation using 3D printed material is a reasonable and cheaper alternative option to practice procedures. Repeated simulations may help increase comfort in performing ED thoracotomy in particular and perhaps invasive procedures in general.

## Figures and Tables

**Table 1a:** (a) Number of residents and their experience

	Level of Training					
	First year		Second year		Third year	
Number of participated residents	8		9		4	

Observed ED thoracotomy	No	Yes	No	Yes	No	Yes

	4	4	7	2	2	2

Participated in ED thoracotomy	No	Yes	No	Yes	No	Yes

	5	3	8	1	3	1

Performed ED thoracotomy	No	Yes	No	Yes	No	Yes

	8	0	9	0	3	1

Watched video demo of ED thoracotomy	No	Yes	No	Yes	No	Yes

	3	5	1	8	0	4

Knowledge of Organs and structures	No	Yes	No	Yes	No	Yes

	0	8	0	9	0	4

Overall Comfort level	Low (1-4)		Moderate (5-7)		High (8-10)	

Initially	15		5		1	

After video demo	10		9		2	

After simulation	With the procedure	Future procedure	With the procedure	Future procedure	With the procedure	Future procedure

	0	0	11	6	10	15

**Table 1b:** (b) Number of residents and their comfort level for each category

	Comfort level			
		Low (1-4)	Moderate (5-7)	High (8-10)
Training level				

First year				

Initially				

	Identifying organs and structures	3	3	2

	If performing procedure now	7	1	0

After video demo				

	Identifying organs and structures	2	2	4

	If performing procedure now	6	2	0

After simulation				

	Identifying organs and structures	0	2	6

	performing procedure	0	4	4

	Performing procedure in the future	0	2	6

Second year				

Initially				

	Identifying organs and structures	3	5	1

	If performing procedure now	7	1	1

After video demo				

	Identifying organs and structures	0	7	2

	If performing procedure now	3	4	2

After simulation				

	Identifying organs and structures	0	1	8

	performing procedure	0	5	4

	Performing procedure in the future	0	3	6

Third year				

Initially				

	Identifying organs and structures	0	4	0

	If performing procedure now	1	3	0

After video demo				

	Identifying organs and structures	0	4	0

	If performing procedure now	1	3	0

After simulation				

	Identifying organs and structures	0	2	2

	performing procedure	0	2	2

	Performing procedure in the future	0	1	3

## Data Availability

The data used to support the findings of this study are available from the corresponding author upon request.

## Supplementary Materials

Supplementary Materials Supplement 1: Questionnaire pretraining. Supplement 2: Questionnaire presimulation training: Video demonstration. Supplement 3: questionnaire posttraining.
