# Supplementary material for: Development and Utilization of 3D Printed Material for Thoracotomy Simulation
Source: Emerg Med Int. 2018 Nov 15;2018:9712647. doi: 10.1155/2018/9712647 (PMC6276476; doi:10.1155/2018/9712647)
Supplement: Supplementary Materials — Supplement 1: Questionnaire pretraining. Supplement 2: Questionnaire presimulation training: Video demonstration. Supplement 3: questionnaire posttraining. [file 9712647.f1.pdf]

Supplements

Supplement 1

Questionnaire pre-training:

1. What is your PGY level?
2. Have you performed an ED thoracotomy?
3. If yes, how many have you performed?
4. Have you participated in ED thoracotomy?
5. Have you observed an ED thoracotomy?
6. Have you watched videos of an ED thoracotomy?
7. On a scale from 1-10 (1 being not comfortable, 10 being very comfortable), how do you rate your current level of comfort in performing an ED thoracotomy?
8. Do you know the organs in the left chest cavity?
9. On a scale from 1-10 (1 being not comfortable, 10 being very comfortable), how do you rate your current level of ability to accurately identify anatomical structures in the left chest cavity?

Supplement 2

Questionnaire pre-training: Video

1. What is your PGY level?
2. On a scale from 1-10 (1 being not comfortable, 10 being very comfortable), how do you rate your current level of comfort in performing an ED thoracotomy after the demo?
3. On a scale from 1-10 (1 being not comfortable, 10 being very comfortable), how do you rate your current level of ability to accurately identify anatomical structures in the left chest cavity?

Supplement 3

Questionnaire post-training:

1. What is your PGY level?
2. On a scale from 1-10 (1 being not comfortable, 10 being very comfortable), how do you rate your current level of comfort in performing the ED thoracotomy?
3. On a scale from 1-10 (1 being not comfortable, 10 being very comfortable), how do you rate your current level of ability to accurately identify anatomical structures in the left chest cavity?
4. On a scale from 1-10 (1 being not comfortable, 10 being very comfortable), how do you rate your comfort level in your ability to perform this procedure again?
